# Relationship between sRAGE and obesity in individuals with type 1 diabetes during a median follow-up of 6.3 years

**DOI:** 10.1007/s00125-025-06440-4

**Published:** 2025-04-29

**Authors:** Krishna Adeshara, Erika B. Parente, Valma Harjutsalo, Markku Lehto, Niina Sandholm, Per-Henrik Groop, on behalf of the FinnDiane Study Group

**Affiliations:** 1 Folkhälsan Research Center, Helsinki, Finland; 2 Research Program for Clinical and Molecular Metabolism, University of Helsinki, Helsinki, Finland; 3 Department of Nephrology, University of Helsinki and Helsinki University Hospital, Helsinki, Finland; 4 Boehringer Ingelheim International GmbH, Ingelheim, Germany; 5 Department of Diabetes, Central Clinical School, Monash University, Melbourne, VIC Australia; 6 Baker Heart and Diabetes Institute, Melbourne, VIC Australia

**Keywords:** BMI, Diabetes, Kidney disease, Obesity, sRAGE, WHtR

## Abstract

**Aims/hypothesis:**

Soluble receptor for advanced glycation end products (sRAGE) has been inversely linked to obesity, which is defined by excess of total body fat. However, body fat accumulation is also relevant for health. In this study, we investigated associations between sRAGE and obesity in individuals with type 1 diabetes over 6.3 years of follow-up.

**Methods:**

The study included 3886 adults with type 1 diabetes from the FinnDiane study. Serum sRAGE concentrations were determined by ELISA. Central obesity was defined on the basis of waist/height ratio (WHtR), and general obesity on the basis of BMI. The Kruskal–Wallis test was used to assess the differences in baseline BMI, WHtR and sRAGE concentrations, comparing the groups stratified by albuminuria status. Changes in BMI and WHtR were calculated over time and Wilcoxon rank test was used for comparisons. Linear regression, adjusted for sex, age, albuminuria and HbA_1c_, was used for assessing the association of sRAGE with obesity measures at baseline, and with changes over time.

**Results:**

Over a median follow-up of 6.3 years, BMI changed by a median Δ of 0.76 kg/m^2^ (IQR −0.39 to 2.07; *p*<0.001) and WHtR by a median Δ of 0.019 (IQR −0.007 to 0.05; *p*<0.001). The change in BMI was observed in 67% of the individuals, and WHtR in 68% of them. Baseline sRAGE was inversely associated with BMI (*r*^2^=0.07, β −0.174; *p*<0.001) and WHtR (*r*^2^=0.16, β −0.179; *p*<0.001) in the overall cohort. These relationships remained consistent across subgroups stratified by albuminuria status, including no, moderate and severe albuminuria (all *p*<0.001). However, sRAGE was not associated with changes in BMI or WHtR over time.

**Conclusions/interpretation:**

sRAGE is inversely associated with both general and central obesity, as represented by BMI and WHtR, independent of kidney disease, suggesting sRAGE is a biomarker of obesity. However, sRAGE is not associated with the changes in BMI and WHtR over a 6.3 year follow-up. Future research with longer follow-up is merited to understand how sRAGE correlates with body fat accumulation.

**Graphical Abstract:**

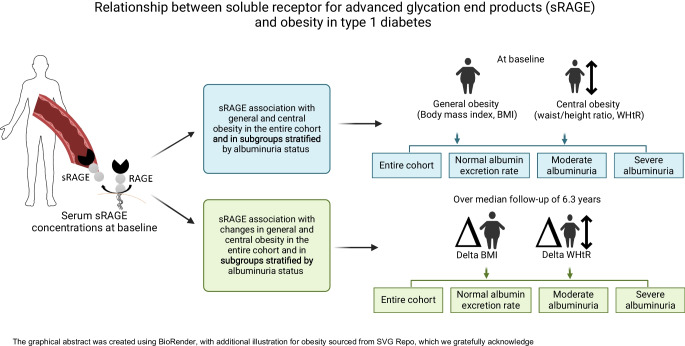

**Supplementary Information:**

The online version of this article (10.1007/s00125-025-06440-4) contains peer-reviewed but unedited supplementary material.


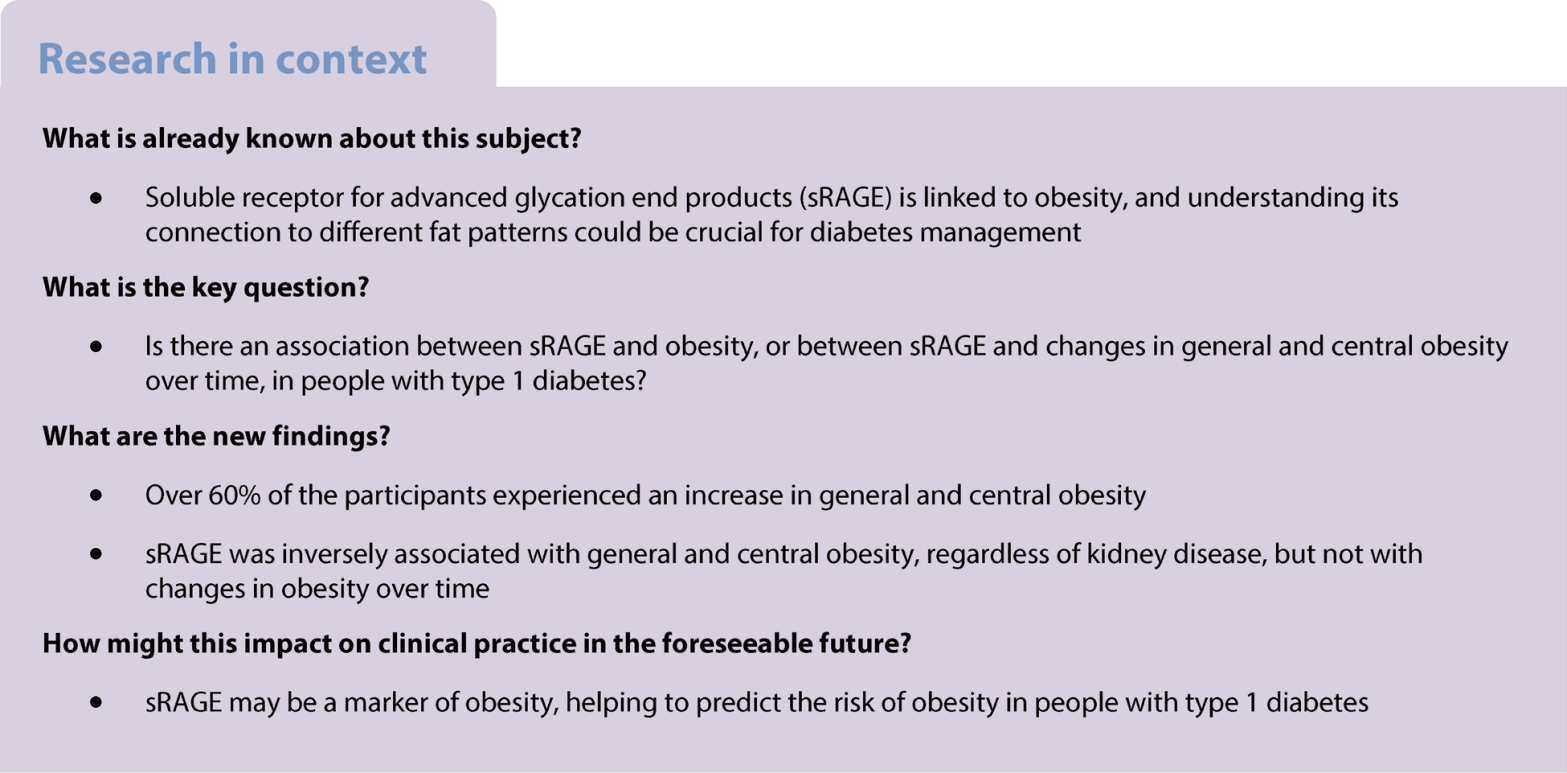


## Introduction

The receptor for advanced glycation end products (RAGE) has been associated with obesity. It is expressed in various cells such as adipocytes, endothelial cells and macrophages. There are two isoforms of RAGE: one is the full-length surface RAGE, which is capable of inducing intracellular signalling; and the other is a truncated isoform called soluble RAGE (sRAGE). The sRAGE is a mixture of cleaved RAGE (cRAGE) produced via proteolytic shedding of the RAGE ectodomain, and endogenous secretory RAGE (esRAGE) generated by alternative splicing of RAGE pre-RNA [1].

Ligand interaction with surface RAGE triggers NF-κB-mediated cytokine secretion and production of reactive oxygen species. The sRAGE isoform circulates in the blood, acting as a decoy receptor for AGEs, thereby inhibiting the AGE–RAGE interaction and modulating inflammation [2]. The modulatory effect of sRAGE may influence body fat composition and obesity. A meta-analysis of cross-sectional studies showed that apparently healthy individuals with higher sRAGE concentrations had lower BMI and waist circumference [3]. Other studies showed that lower sRAGE concentrations were not only associated with visceral fat accumulation at the epicardial level in healthy adult women [4] but also correlated with higher BMI and waist circumference in women living with obesity [5] and with adiposity in young adults without diabetes [6]. Interestingly, one study found that in individuals living with obesity, those who had lower baseline sRAGE concentrations experienced greater weight loss and improved their insulin sensitivity after 12 weeks on a diet very low in energy (very low-calorie diet [VLCD]) compared with those who had higher baseline sRAGE concentrations [7]. An experimental study in mice also showed an inverse association of sRAGE with BMI, insulin resistance and body weight gain [8]. Thus, the observed lower sRAGE concentrations seen in obesity may reflect greater binding of AGEs to their ligands, thereby promoting inflammation [2].

sRAGE is an important biomarker of kidney disease. Our group has reported elevated baseline sRAGE concentrations in individuals with diabetic nephropathy, and its association with the progression from macroalbuminuria to end-stage kidney disease [9]. AER is a more sensitive and earlier indicator of kidney damage than the decline in eGFR [10]. A study by Humpert et al showed that sRAGE is positively correlated with the 24 h albumin excretion in individuals with type 2 diabetes and suggested that sRAGE could be a marker of early diabetic kidney disease in these individuals [11]. Therefore, assessing sRAGE in the context of AER could potentially help to monitor kidney health and track disease progression.

Obesity is defined by excessive accumulation of body fat and a BMI of ≥30 kg/m^2^. However, beyond BMI, central fat mass is of utmost importance for health [12]. In previous studies, we explored indicators of central obesity and observed that waist/height ratio (WHtR) was the best predictor of central body fat [13]. Furthermore, we showed that WHtR was more strongly associated with several diabetes complications, such as metabolic dysfunction-associated steatotic liver disease [14], severe diabetic eye disease [15], onset of albuminuria [16] and hospitalisation and death due to heart failure [17], than BMI in individuals with type 1 diabetes.

Though studies in the general population have mostly shown an inverse relationship between sRAGE and obesity, it remains unclear whether sRAGE can serve as an indicator of obesity in individuals with type 1 diabetes, including general and central obesity. Furthermore, we hypothesise that baseline sRAGE concentrations might influence the accumulation of body fat over time. In the present study, we aimed to investigate the relationship between sRAGE and obesity represented by two different types, general obesity and central obesity, in the entire cohort as well as in subgroups stratified by albuminuria status. In addition, we investigated whether sRAGE is associated with changes in general and central obesity during a follow-up of 6.3 years in individuals with type 1 diabetes.

## Methods

### Study participants

This Finnish Diabetic Nephropathy (FinnDiane) Study cohort included adults diagnosed with type 1 diabetes by their attending physician (*N*=3886), with data collected between January 1998 and November 2013. A subset of these individuals (*n*=1610) also had BMI and WHtR data available from a follow-up visit. Type 1 diabetes was further defined as age at diabetes onset below 40 years, and insulin treatment initiated within 1 year of the diagnosis. A more detailed description of the study cohort is given elsewhere [18, 19]. The study protocol follows the Declaration of Helsinki and was approved by the Ethics Committee of the Helsinki and Uusimaa Hospital District (HUS) (491/E5/2006, 238/13/03/00/2015 and HUS-3313-2018, 3 July 2019). All participants gave their informed written consent. The study included a balanced representation of the general population, comprising 51% male and 49% female participants, all of European ethnicity. The participants had an average age of 38 years and were predominantly Finnish individuals residing in Finland. The participants’ socioeconomic status was based on education and professional status. The participants’ sex was assigned by the attending physician or study nurse. Individuals with missing baseline anthropometric data and sRAGE measurements were excluded from the study. Additionally, individuals undergoing kidney replacement therapy (KRT), defined as the participant being on dialysis treatment, or who had received a kidney transplant at baseline, were excluded.

### Albuminuria status

Participants were requested to collect 24 h urine samples, and two additional timed overnight urine samples. The AER in the 24 h urine sample was measured centrally by a photometric immunochemical method (Siemens Healthineers, Forchheim, Germany), while the AER in the overnight urine samples was measured locally by standardised methods. In addition, AER values preceding the study visit were collected from the participants’ medical records. Normal urinary AER was defined as AER <20 μg/min or <30 mg/24 h, moderate albuminuria as AER ≥20 and <200 μg/min or ≥30 and <300 mg/24 h, and severe albuminuria as AER ≥200 μg/min or ≥300 mg/24 h. This classification was based on the agreement of at least two out of three consecutive urine collections. The presence of albuminuria was defined as moderate or severe albuminuria.

### Clinical measurements and laboratory analysis

Blood samples were collected for the analysis of HbA_1c_, lipids and plasma creatinine. HbA_1c_ and plasma creatinine concentrations were measured locally by standardised assays. Serum lipid concentrations were all measured centrally from serum samples using previously described methods [20]. LDL-cholesterol was calculated with the Friedewald formula if triglycerides were <4.0 mmol/l [21]. BP was measured twice by a trained nurse at a 2 min interval in the sitting position with a 10 min rest before the first measurement; the mean value of these two measurements was calculated for both the systolic BP (SBP) and the diastolic BP (DBP). BP measurement was done using a mercury sphygmomanometer or an automated standardised BP device (OMRON M6 BP monitor, Omron Electronics, Espoo, Finland). sRAGE concentrations were measured from serum samples collected and stored at baseline. The measurements were performed in triplicate using a commercial ELISA (DRG00; R&D Systems, Minneapolis, MN, USA), according to the manufacturer’s instructions at the FinnDiane Research Laboratory.

### Anthropometric measures

BMI was calculated by total body weight (in kg) divided by the square of height (m^2^), and individuals with BMI ≥30 kg/m^2^ were considered to have general obesity. A stretch-resistant tape measure was used to measure the waist circumference at the horizontal plane midway of the superior iliac crest and the lower margin of the last rib. The WHtR was calculated by dividing the waist circumference by the height, and a WHtR ≥0.5 defined central obesity, independent of sex.

### Statistical analyses

Data on categorical variables are presented as frequencies, while continuous variables are shown as means ± SD if normally distributed and as median (IQR) if distribution was skewed. Changes in BMI and WHtR over time were calculated by the difference (Δ) between the baseline and the end of follow-up. Wilcoxon signed-rank test was used to compare BMI and WHtR over time. Mann–Whitney *U* test was used to compare baseline sRAGE concentrations between individuals with and without general obesity, as well as between those with and without central obesity. Kruskal–Wallis test, accompanied by Dunn’s post hoc analyses for pairwise comparisons, with Bonferroni correction for multiple comparisons was used to assess the differences in baseline BMI, WHtR and sRAGE concentrations between the groups stratified by albuminuria status. Similarly, the same tests were applied to assess changes in BMI (ΔBMI) and WHtR (ΔWHtR) across the groups. At baseline, to evaluate the associations between sRAGE and obesity, represented by general and central obesity, we used a linear regression model adjusted for sex, baseline age, presence of albuminuria and HbA_1c_ when applicable. The model was applied to the entire cohort as well as to the subgroups stratified by albuminuria status. Further, we used a similar linear regression model for the associations between sRAGE and ΔBMI and ΔWHtR over time. A two-tailed *p* value of <0.05 was considered statistically significant. Analyses with the Kruskal–Wallis test were conducted using R (version 4.3.3; 2024-02-29 ucrt, https://cran.rstudio.com/). All other analyses were performed using IBM SPSS Statistics for Windows 29 software (IBM Corp., Armonk, NY, USA), unless otherwise stated.

## Results

### Clinical characteristics of the study participants

This study comprised 3886 adults with type 1 diabetes from the FinnDiane study cohort. The median (IQR) age at baseline was 37.5 years (28.2–47.5) and the median (IQR) duration of diabetes was 19.2 years (10.3–29.3); 51% were men. At baseline, median (IQR) measurements were HbA_1c_ 67.2 mmol/mol (57.4–77.0) (8.3% [7.4–9.2]), BMI 24.8 kg/m^2^ (22.7–27.0), WHtR 0.49 (0.46–0.54) and sRAGE 1138 pg/ml (865–1474). At baseline, 9.0% (*n*=351) of the individuals presented with general obesity and 44.7% (*n*=1738) with central obesity. Regarding the albuminuria status, 71.6% of the individuals had normal AER, 14.1% had moderate albuminuria and 14.2% had severe albuminuria. The clinical characteristics of the participants at baseline are depicted in Table 1.

**Table 1:** Baseline clinical characteristics of study participants

Characteristic	Measurement
*N*	3886
Age, years	37.5 (28.2–47.5)
Women, *n* (%)	1885 (49)
Duration of diabetes, years	19.2 (10.3–29.3)
BMI, kg/m^2^	24.8 (22.7–27.0)
General obesity, *n* (%)	351 (9.03)
WHtR	0.49 (0.46–0.54)
Central obesity, *n* (%)	1738 (44.72)
Systolic BP, mmHg	133 ± 17
Diastolic BP, mmHg	79 ± 10
Total cholesterol, mmol/l	4.83 (4.25–5.45)
HDL-cholesterol, mmol/l	1.30 (1.09–1.57)
LDL-cholesterol, mmol/l	2.95 (2.44–3.56)
Triglycerides, mmol/l	1.01 (0.76–1.42)
HbA_1c_, mmol/mol	67.2 (57.4–77.0)
HbA_1c_, %	8.3 (7.4–9.2)
sRAGE, pg/ml	1138 (865–1474)
Normal AER, *n* (%)	2784 (71.6)
Moderate albuminuria, *n* (%)	549 (14.1)
Severe albuminuria, *n* (%)	553 (14.2)

Data on categorical variables are presented as *n* (%), while continuous variables are shown as means ± SD for normal distribution and as median (IQR) for skewed distribution

Individuals with general obesity had lower sRAGE concentrations at baseline compared with their counterparts without general obesity (median [IQR]: 1019 pg/ml [760–1366] vs 1147 pg/ml [874–1485], *p*<0.001). Similarly, individuals with central obesity had lower sRAGE concentrations at baseline compared with those without central obesity (median [IQR]: 1060 pg/ml [801–1380] vs 1194 pg/ml [923–1557], *p*<0.001). Further, these individuals with general or central obesity at baseline were stratified based on albuminuria status. sRAGE concentrations were elevated in the individuals with severe albuminuria compared with those with normal AER, regardless of whether they had general or central obesity at baseline (data not shown). These data show that kidney disease itself may contribute to the changes in sRAGE concentrations, independent of obesity.

In our previous work [9] we demonstrated that baseline sRAGE concentrations were higher in individuals with diabetic nephropathy than in those with normal AER or microalbuminuria, and also that the elevated sRAGE concentrations were linked to the progression from macroalbuminuria to end-stage kidney disease. In the present study, we assessed the sRAGE concentrations and the obesity variables across the groups categorised by albuminuria status as well as the relationship of sRAGE with general and central obesity. Based on albuminuria groups, individuals with moderate and severe albuminuria had significantly higher BMI and WHtR levels compared with those without albuminuria. Median (IQR) BMI was 25.5 kg/m^2^ (23.1–28.0) vs 24.5 kg/m^2^ (22.5–26.6) (*p*<0.001) and median (IQR) WHtR was 0.51 (0.47–0.55) vs 0.48 (0.45–0.52) (*p*<0.001) in those with moderate albuminuria vs those without albuminuria, respectively. For participants with severe albuminuria vs those without albuminuria, respectively, the median BMI (IQR) was 25.7 kg/m^2^ (23.3–28.5) vs 24.5 kg/m^2^ (22.5–26.6) (*p*<0.001) and the median (IQR) WHtR was 0.52 (0.48–0.57) vs 0.48 (0.45–0.52) (*p*<0.001). Notably, WHtR was modestly higher in the severe albuminuria than in the moderate albuminuria group (*p*<0.01), whereas no significant difference was seen in BMI between the moderate and severe albuminuria groups. Furthermore, the sRAGE concentrations were significantly higher in participants with severe albuminuria (median 1371 pg/ml [IQR 1003–1897]) than in participants without albuminuria (median 1114 pg/ml [IQR 858–1426], *p*<0.001) or participants with moderate albuminuria (median 1079 pg/ml [IQR 805–1394], *p*<0.001). However, there were no differences in sRAGE concentrations between the moderate albuminuria group and those without albuminuria.

### Change in BMI and WHtR over time

**Fig. 1:**
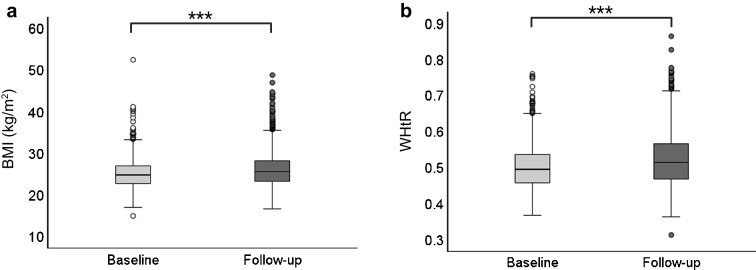
Changes in general obesity (**a**, BMI) and central obesity (**b**, WHtR) over time. Data are shown as median (IQR) of BMI and WHtR at baseline and after 6.3 years of follow-up (*n*=1610). Wilcoxon rank test was used to compare medians from baseline to end of follow-up time. ***p<0.001. The whiskers in the boxplots represent the range of the data, excluding potential outliers. Specifically, they extend to the smallest and largest values within 1.5 times the IQR from the first and third quartiles, respectively. The data points outside these whiskers are outliers

For individuals with anthropometric follow-up data available (*n*=1610), during a median follow-up of 6.3 years (IQR 5.0–7.9), BMI increased from a median (IQR) of 24.8 kg/m^2^ (22.8–27.0) to 25.6 kg/m^2^ (23.4–28.3) (*p*<0.001) and WHtR increased from 0.49 (0.46–0.54) to 0.51 (0.47–0.57) (*p*<0.001) (Fig. 1). The median ΔBMI was 0.76 kg/m^2^ (IQR −0.39 to 2.07) and the median ΔWHtR was 0.019 (IQR −0.007 to 0.047). Overall, BMI increased in 67% (*n*=1083) of the individuals and WHtR increased in 68% (*n*=1093).

Changes in BMI and WHtR over time, stratified by albuminuria groups, are illustrated in ESM Fig. 1. Among individuals without albuminuria, BMI increased in 68% (*n*=803) and WHtR in 68% (*n*=798). In the moderate albuminuria group, BMI increased in 66% (*n*=151) and WHtR increased in 68% (*n*=157) of the individuals. For those with severe albuminuria, 63% (*n*=129) experienced an increase in BMI and 68% (*n*=138) had an increase in WHtR.

Notably, the median (IQR) ΔBMI differed between those without albuminuria and those with severe albuminuria (0.76 kg/m^2^ [−0.33–2.07] vs 0.49 kg/m^2^ [−0.72 to 1.93], *p*=0.04), while no significant difference was observed in ΔBMI when the moderate albuminuria group was compared either with the severe albuminuria or the normal AER group. Additionally, no significant differences were observed in ΔWHtR between any of the albuminuria groups. The clinical characteristics of the participants included in the follow-up analysis are presented in ESM Table 1. Notably, no differences were observed in the clinical variables of this subset compared with the overall cohort.

### Associations between sRAGE, BMI and WHtR

At baseline, sRAGE was inversely associated with general obesity, represented by BMI (*r*^2^=0.07, β −0.174, *p*<0.001), and with central obesity, represented by WHtR (*r*^2^=0.16, β −0.179, *p*<0.001) (Table 2) in the entire cohort of individuals with type 1 diabetes. In the subgroup analysis by albuminuria status, these inverse associations persisted across all groups, including the groups without albuminuria, with moderate albuminuria and with severe albuminuria, for both BMI (*p*<0.001) and WHtR (*p*<0.001) (Table 2). However, no significant associations were found between baseline sRAGE and the ΔBMI and ΔWHtR over time in either the whole cohort or any of the albuminuria subgroups (Table 3).

**Table 2:** Baseline associations between sRAGE and BMI, and between sRAGE and WHtR

Variable	Unadjusted model			Adjusted model		
	*r*^2^	Standardised coefficient (β)	*p* value	*r*^2^	Standardised coefficient (β)	*p* value
BMI						
All	0.02	−0.141	0.001	0.07	−0.174	0.001
Normal AER	0.03	−0.176	0.001	0.05	−0.169	0.001
Moderate albuminuria	0.05	−0.218	0.001	0.06	−0.212	0.001
Severe albuminuria	0.03	−0.166	0.001	0.05	−0.173	0.001
WHtR						
All	0.02	−0.131	0.001	0.16	−0.179	0.001
Normal AER	0.04	−0.202	0.001	0.12	−0.188	0.001
Moderate albuminuria	0.06	−0.248	0.001	0.11	−0.225	0.001
Severe albuminuria	0.02	−0.142	0.001	0.13	−0.152	0.001

The analysis included all individuals with measures of BMI, WHtR and sRAGE at baseline visit (*n*=3886). Subgroup analyses were performed separately in participants with normal AER (*n*=2784), moderate albuminuria (*n*=549) and severe albuminuria (*n*=553)

The linear regression models were adjusted for sex, baseline age, the presence of albuminuria and HbA_1c_; the analysis in normal, moderate and severe albuminuria groups were adjusted for sex, baseline age and HbA_1c_ only

**Table 3:** Associations between baseline sRAGE and ΔBMI and ΔWHtR over 6.3 years of follow-up

Variable	Unadjusted model			Adjusted model		
	*r*^2^	Standardised coefficient (β)	*p* value	*r*^2^	Standardised coefficient (β)	*p* value
BMI						
All	0.0003	−0.016	0.511	0.05	−0.019	0.455
Normal AER	0.002	0.042	0.147	0.05	0.035	0.215
Moderate albuminuria	0.008	−0.088	0.182	0.05	−0.092	0.158
Severe albuminuria	0.007	−0.082	0.245	0.04	−0.103	0.143
WHtR						
All	0.000001	−0.001	0.966	0.02	−0.008	0.745
Normal AER	0.001	0.033	0.265	0.02	0.029	0.315
Moderate albuminuria	1.16×10^-7^	−0.0003	0.996	0.04	−0.001	0.984
Severe albuminuria	0.006	−0.080	0.257	0.06	−0.097	0.162

The analysis included all individuals with measures of BMI and WHtR at baseline and follow-up visits (*n*=1610). Subgroup analyses were performed separately in participants with normal AER (*n*=1176), moderate albuminuria (*n*=230) and severe albuminuria (*n*=204)

The linear regression models were adjusted for sex, baseline age, the presence of albuminuria and HbA_1c_; the analysis in normal, moderate and severe albuminuria groups were adjusted for sex, baseline age and HbA_1c_ only

As a sensitivity analysis, we repeated the analyses stratified by sex. sRAGE remained negatively associated with BMI and WHtR in both male and female participants with type 1 diabetes when adjusted for age, albuminuria status and HbA_1c_ in the full cohort (*p*<0.001), and in all subgroups divided by albuminuria (*p*<0.05). No significant associations (*p*>0.05) were observed between baseline sRAGE concentrations and ΔBMI or ΔWHtR over time, either in the entire cohort or within the albuminuria subgroups, for both male and female participants.

## Discussion

This study demonstrated an inverse relationship between sRAGE concentrations and both general and central obesity, regardless of the presence or severity of albuminuria. However, sRAGE was not associated with any changes in general or central obesity over time, suggesting that sRAGE is a mere biomarker of obesity, rather than a key player in body fat changes. So far, this is the first study to explore the relationship between sRAGE concentrations and obesity, represented by BMI and WHtR, in people living with type 1 diabetes with or without kidney disease.

sRAGE is known to act as a decoy receptor, which can bind to AGEs and other proinflammatory ligands, further preventing their binding to surface RAGE. This neutralisation reduces the activation of proinflammatory signalling and potentially acts as a protective factor against systemic inflammation. Studies have shown contradictory findings regarding plasma concentration of sRAGE and different metabolic diseases. High concentrations of sRAGE have been observed in individuals with type 2 diabetes [22], in people with type 1 diabetes and diabetic nephropathy [9], in people with diabetes and coronary artery disease [23] and even in the healthy general population [24]. In contrast, the Northern Manhattan Study, a study in the general population, showed that lower concentrations of sRAGE were associated with components of the metabolic syndrome, including elevated BMI and central obesity [25]. Lower concentrations of sRAGE were observed also in individuals with impaired glucose tolerance and type 2 diabetes [26], as well as in individuals living with obesity compared with their lean counterparts [4, 27]. In our study, we also observed an inverse association between sRAGE and obesity in the cohort of people with type 1 diabetes. Moreover, we showed that the association is present regardless of body fat accumulation and severity of albuminuria. Additionally, participants with severe albuminuria exhibited elevated sRAGE concentrations, indicating that kidney disease might play a role in modifying the sRAGE concentrations. One explanation for the contradictory results in the literature may involve the differences in populations as well as differences in health conditions. Due to its observational design, the present study cannot explain these differences, and future mechanistic studies are necessary to better understand the observed differences in the sRAGE concentrations in various disease conditions.

sRAGE has been associated with obesity and its variations may reflect the metabolic dysregulation associated with excessive adiposity [4, 7]. Of note, we observed a higher prevalence of central obesity than general obesity in our cohort, highlighting the relevance of measuring central obesity beyond BMI, as central obesity is more strongly associated than BMI with diabetes complications such as severe eye disease [15], fatty liver [13], albuminuria onset [16] and hospitalisation and death due to heart failure [17]. In the present study, sRAGE was also inversely associated with obesity. While we showed in our previous studies that obesity is associated with albuminuria onset or increased cardiovascular mortality risk [28] and that elevated sRAGE is also associated with diabetic kidney disease or cardiovascular mortality risk [9, 29], the current results suggest that the liaisons between sRAGE and diabetes complications are not via obesity but either reflect underlying obesity or are mediated through other, obesity-independent pathways.

Obesity trajectory has been linked to the risk of chronic complications such as cardiometabolic diseases and diabetes. For instance, a study in the general population from Norway reported that change in BMI over 2 years was associated with the increased risk of hypertension in men [30]. Similarly, a Japanese study reported that changes in BMI over 9 years were associated with changes in BP [31] and a Chinese study showed that weight gain from young to middle adulthood was associated with an increased risk of mortality [32]. Furthermore, high WHtR and waist circumference, which represent central obesity, have been linked to increased risk of cardiometabolic diseases [33], higher risk of all-cause mortality [34] and risk of multimorbidity [35]. Our current study showed a significant increase in general obesity (BMI), and particularly central obesity (WHtR), in more than 60% of the participants and a similar pattern was observed in those with moderate or severe albuminuria. Interestingly, the change in BMI (ΔBMI) was significantly different between the normal AER and the severe albuminuria groups, with a more pronounced change observed in those without albuminuria. Furthermore, the cohort comprises 72% of individuals with normal AER and only 14% with severe albuminuria. This distribution may explain the more pronounced change in ΔBMI observed among the individuals with normal AER. This finding suggests that kidney disease may be linked to the progression of BMI and that general obesity could be an important predictor of early kidney disease. However, these results need to be replicated in a larger cohort to confirm their validity. Additionally, we did not find any associations between sRAGE and changes in BMI and WHtR in our study. Therefore, our findings suggest that sRAGE is simply a biomarker of obesity.

Aligned with this conclusion and with our findings, other studies have shown an inverse association between sRAGE and BMI [36, 37], waist circumference and visceral adipose tissue [4]. Furthermore, similar to our results, Kanikowska et al reported no association between baseline sRAGE and weight loss after 2 months of energy restriction in individuals living with obesity [38]. Additionally, Popp et al showed no associations between baseline sRAGE and changes in body composition [39]. These findings suggest that although sRAGE is a marker of inflammation, it may not be able to predict long-term metabolic changes in obesity.

In our study, sex-stratified analysis did not reveal any significant differences in the association between sRAGE and obesity. sRAGE remained negatively associated with BMI and WHtR in both male and female participants across the entire cohort, as well as within albuminuria subgroups. However, sRAGE showed no association with changes in obesity over time in either sex. Therefore these findings may be broadly applicable to both male and female individuals. Nevertheless, as the study was conducted within a European population, the results may not be generalised to populations of other ethnicities.

The study has some limitations. We measured a pool of total serum sRAGE, which cannot distinguish between the isoforms resulting from ectodomain shedding (cRAGE) or alternative splicing (esRAGE). These isoforms have distinct functions and mechanisms of action. cRAGE primarily reflects the state of inflammation, while esRAGE is influenced by genetic variation [40]. Furthermore, we cannot exclude a possible influence of diet and medication on the sRAGE concentrations and the observed associations. The study also has some strengths. It is noteworthy that this is the first prospective study evaluating the relationship between sRAGE and body-fat changes in adults with type 1 diabetes. Furthermore, it includes a large cohort of well-characterised participants with type 1 diabetes, prospective study design with longer follow-up visits and available data on diabetes complications.

In conclusion, sRAGE is inversely associated with both general and central obesity, as represented by BMI and WHtR, independent of kidney disease, suggesting that sRAGE is a biomarker of obesity. However, it shows no association with the changes in BMI and WHtR over 6.3 years of follow-up in adults with type 1 diabetes. Future research is merited with longer follow-up to understand how sRAGE correlates with body fat accumulation.

## Supplementary Information

Below is the link to the electronic supplementary material.

ESM (PDF 181 KB)

## Data Availability

Individual-level data for the study participants are not publicly available because of the restrictions due to the study consent provided by the participant at the time of data collection.

## Abbreviations

cRAGE: Cleaved RAGE
DBP: Diastolic BP
esRAGE: Endogenous secretory RAGE
FinnDiane: Finnish Diabetic Nephropathy study
RAGE: Receptor for advanced glycation end products
SBP: Systolic BP
sRAGE: Soluble RAGE
WHtR: Waist/height ratio

## Authors’ relationships and activities

P-HG has received research grants from Eli Lilly and Roche, and lecture fees from Astellas, Astra Zeneca, Bayer, Berlin Chemie, Boehringer Ingelheim, Eli Lilly, Elo Water, Genzyme, Medscape, Menarini, MSD, Mundipharma, Novartis, Novo Nordisk, PeerVoice, Sanofi and SCIARC. He is an advisory board member for AbbVie, Astellas, Bayer, Boehringer Ingelheim, Eli Lilly, Janssen, Medscape, MSD, Mundipharma, Nestlé, Novartis, Novo Nordisk and Sanofi. EBP reports receiving lecture honorariums from Astra Zeneca and Sanofi, and has been an employee of Boehringer Ingelheim since February 2024. The authors declare that there are no other relationships or activities that might bias, or be perceived to bias, their work.
