# Supplementary material for: Relationship between sRAGE and obesity in individuals with type 1 diabetes during a median follow-up of 6.3 years
Source: Diabetologia. 2025 Apr 29;68(8):1657–65. doi: 10.1007/s00125-025-06440-4 (PMC12246012; doi:10.1007/s00125-025-06440-4)
Supplement: Supplementary file 1 — ESM (PDF 181 KB) [file 125_2025_6440_MOESM1_ESM.pdf]

ESM Table 1. Baseline clinical characteristics of study participants included in follow-up analysis.

n	1610
Age, years	38.5 (29.2, 47.6)
Women, n (%)	805 (50)
Duration of diabetes, years	19.6 (10.4, 30.1)
BMI, kg/m^2	24.8 (22.8, 27.0)
General obesity, n (%)	151 (9.38)
WHtR	0.49 (0.46, 0.54)
Central obesity, n (%)	730 (45.34)
Systolic blood pressure, mmHg	133 ± 17
Diastolic blood pressure, mmHg	79 ± 9.3
Total cholesterol, mmol/L	4.78 (4.25, 5.40)
HDL-cholesterol, mmol/L	1.35 (1.11, 1.59)
LDL-cholesterol, mmol/L	2.91 (2.42, 3.49)
Triglycerides, mmol/L	0.96 (0.73, 1.35)
HbA1c, mmol/mol	66.1 (56.3, 75.9)
HbA1c, %	8.2 (7.3, 9.1)
sRAGE, pg/ml	1124 (864, 1457)
Normal AER, n (%)	1176 (73.0)
Moderate albuminuria, n (%)	230 (14.3)
Severe albuminuria, n (%)	204 (12.7)

Data on categorical variables are presented as frequencies, while continuous variables are shown as means (± standard deviation, SD) if normally distributed and as median (interquartile range, IQR) if skewed distribution. BMI: body mass index, WHtR: waist-height ratio, HDL-cholesterol = high-density lipoprotein cholesterol; LDL-cholesterol = low-density lipoprotein cholesterol; HbA1c: glycated hemoglobin A1c, sRAGE: soluble receptor for advanced glycation end products, AER: albumin excretion rate.

ESM Table 2. Physicians and nurses at each of the FinnDiane centers participating in patient recruitment and characterization

FinnDiane Study Centers	Physicians and nurses
Anjalankoski Health Centre	S. Koivula, T. Uggeldahl
Central Finland Central Hospital, Jyväskylä	T. Forslund, A. Halonen, A. Koistinen, P. Koskiaho, M. Laukkanen, J. Saltevo, M. Tiihonen
Central Hospital of Åland Islands, Mariehamn	M. Forsen, H. Granlund, A-C. Jonsson, B. Nyroos
Central Hospital of Kanta-Häme, Hämeenlinna	P. Kinnunen, A. Orvola, T. Salonen, A. Vähänen
Central Hospital of Länsi-Pohja, Kemi	H. Laukkanen, P. Nyländen, A. Sademies
Central Ostrabothnian Hospital District, Kokkola	S. Anderson, B. Asplund, U. Byskata, P. Liedes, M. Kuusela, T. Virkkala
City of Espoo Health Centre	
Espoonlahti	A. Nikkola, E. Ritola
Tapiola	M. Niska, H. Saarinen
Samaria	E. Oukko-Ruponen, T. Virtanen
Viherlaakso	A. Lyytinen
City of Helsinki Health Centre	
Puistola	H. Kari, T. Simonen
Suutarila	A. Kaprio, J. Kärkkäinen, B. Rantaeskola
Töölö	P. Kääriäinen, J. Haaga, A-L. Pietiläinen
City of Hyvinkää Health Centre	S. Klemetti, T. Nyandoto, E. Rontu, S. Satuli-Autere
City of Vantaa Health Centre	
Korso	R. Toivonen, H. Virtanen
Länsimäki	R. Ahonen, M. Ivaska-Suomela, A. Jauhiainen
Martinlaakso	M. Laine, T. Pellonpää, R. Puranen
Myyrmäki	A. Airas, J. Laakso, K. Rautavaara
Rekola	M. Erola, E. Jatkola
Tikkurila	R. Lönnblad, A. Malm, J. Mäkelä, E. Rautamo
Heinola Health Centre	P. Hentunen, J. Lagerstam
Helsinki University Central Hospital, Department of Medicine, Division of Nephrology	A. Ahola, J. Fagerudd, M. Feodoroff, D. Gordin, O. Heikkilä, K Hietala, L. Kyllönen, J. Kytö, S. Lindh, K. Pettersson-Fernholm, M. Rosengård-Bärlund, M. Rönnback, A. Sandelin, A-R Salonen, L. Salovaara, L. Thorn, J. Tuomikangas, T. Vesisenaho, J. Wadén
Herttoniemi Hospital, Helsinki	V. Sipilä
Hospital of Lounais-Häme, Forssa	T. Kalliomäki, J. Koskelainen, R. Nikkanen, N. Savolainen, H. Sulonen, E. Valtonen
Iisalmi Hospital	E. Toivanen
Jokilaakso Hospital, Jämsä	A. Parta, I. Pirttiniemi
Jorvi Hospital, Helsinki University Central Hospital	S. Aranko, S. Ervasti, R. Kauppinen-Mäkelin, A. Kuusisto, T. Leppälä, K. Nikkilä, L. Pekkonen
Jyväskylä Health Centre, Kyllö	K. Nuorva, M. Tiihonen
Kainuu Central Hospital, Kajaani	S. Jokelainen, P. Kemppainen, A-M. Mankinen, M. Sankari
Kerava Health Centre	H. Stuckey, P. Suominen
Kirkkonummi Health Centre	A. Lappalainen, M. Liimatainen, J. Santaholma
Kivelä Hospital, Helsinki	A. Aimolahti, E. Huovinen
Koskela Hospital, Helsinki	V. Ilkka, M. Lehtimäki
Kotka Heath Centre	E. Pälikkö-Kontinen, A. Vanhanen

FinnDiane Study Centers	Physicians and nurses
Kouvola Health Centre	E. Koskinen, T. Siitonen
Kuopio University Hospital	E. Huttunen, R. Ikäheimo, P. Karhapää, P. Kekäläinen, M. Laakso, T. Lakka, E. Lampainen, L. Moilanen, L. Niskanen, U. Tuovinen, I. Vauhkonen, E. Voutilainen
Kuusamo Health Centre	T. Kääriäinen, E. Isopoussu
Kuusankoski Hospital	E. Kilkki, I. Koskinen, L. Riihelä
Laakso Hospital, Helsinki	T. Meriläinen, P. Poukka, R. Savolainen, N. Uhlenius
Lahti City Hospital	A. Mäkelä, M. Tanner
Lapland Central Hospital, Rovaniemi	L. Hyvärinen, S. Severinkangas, T. Tulokas
Lappeenranta Health Centre	P. Linkola, I. Pulli
Lohja Hospital	T. Granlund, M. Saari, T. Salonen
Loimaa Health Centre	A. Mäkelä, P. Eloranta
Länsi-Uusimaa Hospital, Tammisaari	I-M. Jousmaa, J. Rinne
Malmi Hospital, Helsinki	H. Lanki, S. Moilanen, M. Tilly-Kiesi
Mikkeli Central Hospital	A. Gynther, R. Manninen, P. Nironen, M. Salminen, T. Vänttinen
Mänttä Regional Hospital	I. Pirttiniemi, A-M. Hänninen
North Karelian Hospital, Joensuu	U-M. Henttula, P. Kekäläinen, M. Pietarinen, A. Rissanen, M. Voutilainen
Nurmijärvi Health Centre	A. Burgos, K. Urtamo
Oulankangas Hospital, Oulainen	E. Jokelainen, P-L. Jylkkä, E. Kaarlela, J. Vuolaspuro
Oulu Health Centre	L. Hiltunen, R. Häkkinen, S. Keinänen-Kiukaanniemi
Oulu University Hospital	R. Ikäheimo
Päijät-Häme Central Hospital	H. Haapamäki, A. Helanterä, S. Hämäläinen, V. Ilvesmäki, H. Miettinen
Palokka Health Centre	P. Sopanen, L. Welling
Pieksämäki Hospital	V. Javtsenko, M. Tamminen
Pietarsaari Hospital	M-L. Holmbäck, B. Isomaa, L. Sarelin
Pori City Hospital	P. Ahonen, P. Merensalo, K. Sävelä
Porvoo Hospital	M. Kallio, B. Rask, S. Rämö
Raahe Hospital	A. Holma, M. Honkala, A. Tuomivaara, R. Vainionpää
Rauma Hospital	K. Laine, K. Saarinen, T. Salminen
Riihimäki Hospital	P. Aalto, E. Immonen, L. Juurinen
Salo Hospital	A. Alanko, J. Lapinleimu, P. Rautio, M. Virtanen
Satakunta Central Hospital, Pori	M. Asola, M. Juhola, P. Kunelius, M-L. Lahdenmäki, P. Pääkkönen, M. Rautavirta
Savonlinna Central Hospital	E. Korpi-Hyövälti, T. Latvala, E. Leijala
South Karelia Central Hospital, Lappeenranta	T. Ensala, E. Hussi, R. Härkönen, U. Nyholm, J. Toivanen
Tampere Health Centre	A. Vaden, P. Alarotu, E. Kujansuu, H. Kirkkopelto-Jokinen, M. Helin, S. Gummerus, L. Calonius, T. Niskanen, T. Kaitala, T. Vatanen
Tampere University Hospital	I. Ala-Houhala, T. Kuningas, P. Lampinen, M. Määttä, H. Oksala, T. Oksanen, K. Salonen, H. Tauriainen, S. Tulokas
Tiirismaa Health Centre, Hollola	T. Kivelä, L, Petlin, L. Savolainen
Turku Health Centre	I. Hämäläinen, H. Virtamo, M. Vähätalo
Turku University Central Hospital	K. Breitholz, R. Eskola, K. Metsärinne, U. Pietilä, P. Saarinen, R. Tuominen, S. Äyräpää
Vaajakoski Health Centre	K. Mäkinen, P. Sopanen
Valkeakoski Regional Hospital	S. Ojanen, E. Valtonen, H. Ylönen, M. Rautiainen, T. Immonen
Vammala Regional Hospital	I. Isomäki, R. Kroneld, M. Tapiolinna-Mäkelä
Vaasa Central Hospital	S. Bergkulla, U. Hautamäki, V-A. Myllyniemi, I. Rusk

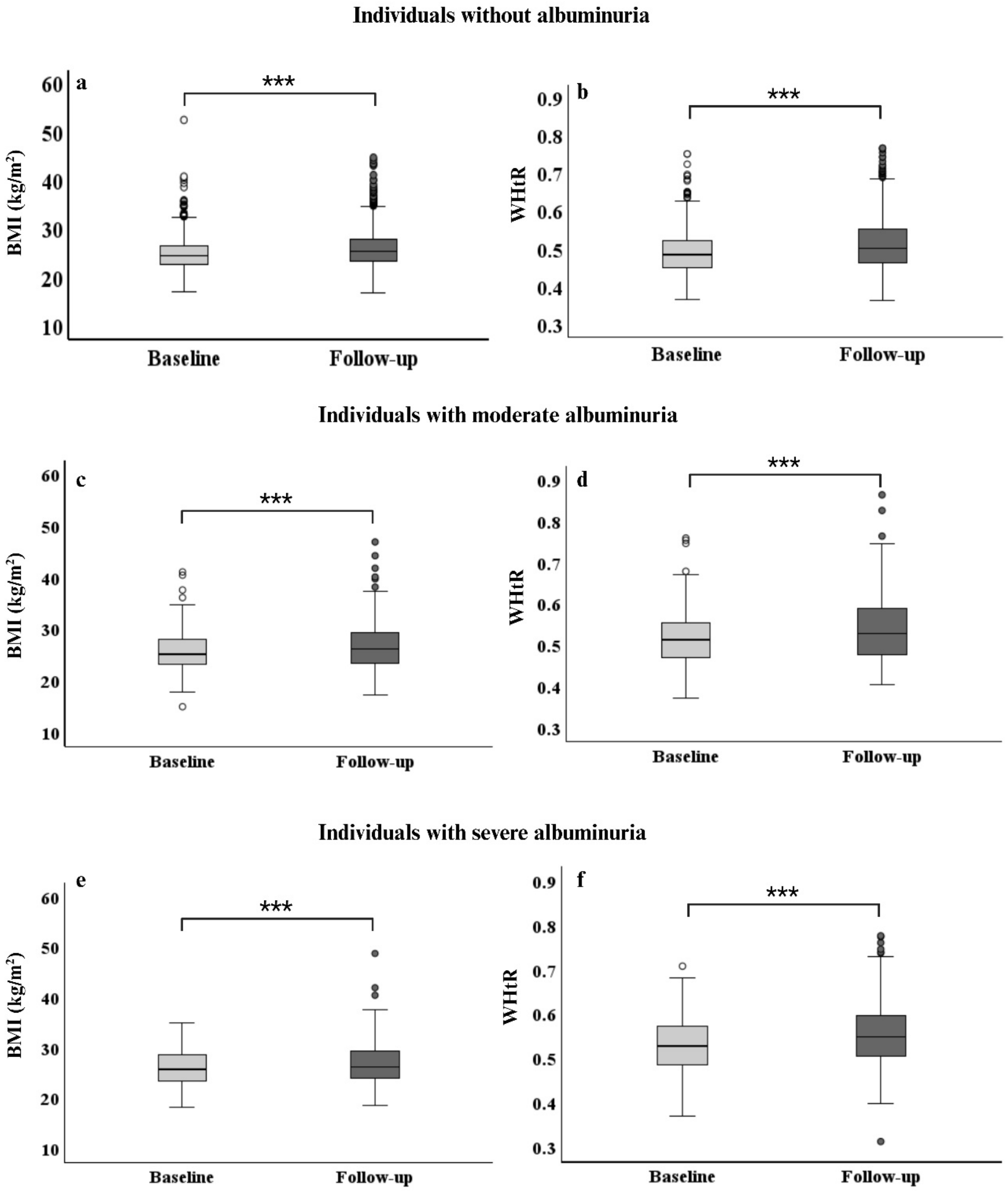

ESM Fig. 1. Changes in general obesity (BMI) and central obesity (WHtR) over time according to albuminuria stages. Data are shown as median (IQR) of BMI and WHtR at baseline and after 6.3 years of follow-up (n=1610). Wilcoxon rank test was used to compare medians from baseline to end follow-up time. Figure a and b represents individuals without albuminuria (n=1176); c and d represents individuals with moderate albuminuria (n=230); e and f represents individuals with severe albuminuria (n=204). ***p<0.001.
